# Identifying multigenic modules under selection in the tumor genome

**DOI:** 10.1093/bioinformatics/btag668

**Published:** 2026-09-09

**Authors:** Marcus R Kelly, Burçak Otlu, Roded Sharan, Trey Ideker

**Affiliations:** School of Medicine, University of California San Diego, La Jolla, CA, United States; Moores Cancer Center, UC San Diego, La Jolla, CA, United States; Moores Cancer Center, UC San Diego, La Jolla, CA, United States; Department of Cellular and Molecular Medicine, UC San Diego, La Jolla, CA, United States; Department of Health Informatics, Graduate School of Informatics, Middle East Technical University, Ankara 06800, Turkey; Blavatnik School of Computer Science and AI, Tel Aviv University, Tel Aviv 6997801, Israel; School of Medicine, University of California San Diego, La Jolla, CA, United States; Moores Cancer Center, UC San Diego, La Jolla, CA, United States; Department of Bioengineering, UC San Diego, La Jolla, CA, United States; Big Data Institute, University of Oxford, Oxford OX26BD, United Kingdom

## Abstract

**Motivation:**

Genomic alterations in cancer arise from selective pressures acting on hallmark molecular modules, layered over a background of random mutagenic events. Methods to detect selection at the level of modules, as opposed to genes or nucleotides, are relatively underdeveloped.

**Results:**

Here we present CanSRMaPP (Cancer Selection Recovery by Maximum Posterior Probability), a Bayesian model of the cancer genome that infers mutational selection on single genes and multi-genic modules while simultaneously modeling background events. Applying CanSRMaPP to lung adenocarcinoma genomes, we identify positive selection on 63 modules, yielding a model that parsimoniously explains the observed pattern of genetic alterations observed in new cancer cohorts. We further show that CanSRMaPP is adaptable to more tumor types and to alternative module definitions. We show that these modules serve as an effective scaffold for translating the cancer genome to molecular states, with prediction of cancer biomarker status as demonstration.

**Availability:**

CanSRMaPP is freely available on GitHub.

## 1 Introduction

A primary goal of cancer genomics is to recognize which genetic alteration events are functional drivers of cancer and which are non-functional “passengers” (Garraway and Lander 2013). Thus far, most approaches to distinguish driver from passenger events have sought to identify genes with frequent alterations, under the premise that high alteration frequency is an indicator of positive selection in tumor cells (Lawrence *et al*. 2013, Mularoni *et al*. 2016, Martincorena *et al*. 2017, Bailey *et al*. 2018, Tokheim and Karchin 2019). Such approaches account for a variety of confounding factors that can influence a gene’s alteration frequency by chance. These factors include gene length, since longer genes naturally accrue more alterations, and replication timing, since early-replicating genes have less time for post-replication DNA repair (Lawrence *et al*. 2013). Furthermore, alteration frequencies are influenced by genome-wide mutagenic processes such as exposure to a specific carcinogen or a specific defect in DNA repair (Garraway and Lander 2013, Alexandrov *et al*. 2020, Steele *et al*. 2022). In this respect, the Catalogue of Somatic Mutations In Cancer (COSMIC) defines signatures for different background mutagenic processes at the level of single- or double-base substitutions (SBS/DBS), short insertions or deletions (ID), or copy number alterations (CN; Ciriello *et al*. 2013b, Alexandrov *et al*. 2020, Steele *et al*. 2022, Otlu *et al*. 2023). Finally, many copy-number alterations can be trivially explained by synteny with nearby genes under direct selection. Thus far, such confounding effects have been combined in multivariate statistical models of cancer genomes, which identify cancer driver genes as those with alteration frequency significantly above that expected after accounting for covariates (Lawrence *et al*. 2013, Mularoni *et al*. 2016, Martincorena *et al*. 2017, Bailey *et al*. 2018, Tokheim and Karchin 2019).

While these approaches have focused on detection of genes, selective forces typically act not on individual factors but on hallmark cancer functions mediated by large modules of interconnected genes (Hanahan and Weinberg 2011). Consequently, the individual genes contributing to each of these functions may be mutated only rarely and thus difficult to detect (Leiserson *et al*. 2015, Zheng *et al*. 2021). Wherever rare mutations converge on a common driving function, however, this function can be more easily identified via a high module-level mutation burden. For example, while the BAF complex as a whole is frequently mutated in many cancer types, the specific mutations involved can differ widely, alternately impacting *ARID1A*, *ARID1B*, *SMARCA2*, and *SMARCA4*, among dozens of complex member genes (Arnaud *et al*. 2018).

To identify the multi-gene modules under selective pressure in cancer, a range of previous approaches have been developed related to active modules (Ideker *et al*. 2002, Chuang *et al*. 2007), network hotspots (Vandin *et al*. 2011, Hofree *et al*. 2013, Leiserson *et al*. 2015, Reyna *et al*. 2020, Yang *et al*. 2021) and mutual exclusivity (Ciriello *et al*. 2013a, Babur *et al*. 2015). These approaches mine a large protein interaction graph to define gene modules (connected sets of genes) that aggregate genetic changes (mutation or gene expression) observed in a cancer subtype. Alternatively, approaches such as gene set enrichment analysis (Subramanian *et al*. 2005, Wendl *et al*. 2011, Paczkowska *et al*. 2020, Li *et al*. 2023) do not identify gene modules de novo but perform independent statistical tests for mutation aggregation within each gene module drawn from a large predefined knowledgebase.

While these approaches have been useful, they are confounded by the fact that gene modules are not independent. Many cancer drivers function pleiotropically within multiple modules, and hierarchical knowledgebases like the Gene Ontology (Ashburner *et al*. 2000) can display a high degree of module overlap and/or redundancy. As a result, mutations to a relatively small number of highly connected genes can produce an outsized number of enriched gene modules, only some of which are under selection in cancer. These challenges motivated our earlier development of HiSig (Zheng *et al*. 2021), a statistical model of mutational selection that analyzes a collection of tumor genomes to infer the distribution of selective pressures across a predefined set of genes and modules. The HiSig model determines whether mutation frequencies observed in a cancer cohort are better explained by many independent selective pressures acting on individual genes, or by fewer selective pressures acting on a small number of multi-gene modules.

**Figure 1:**
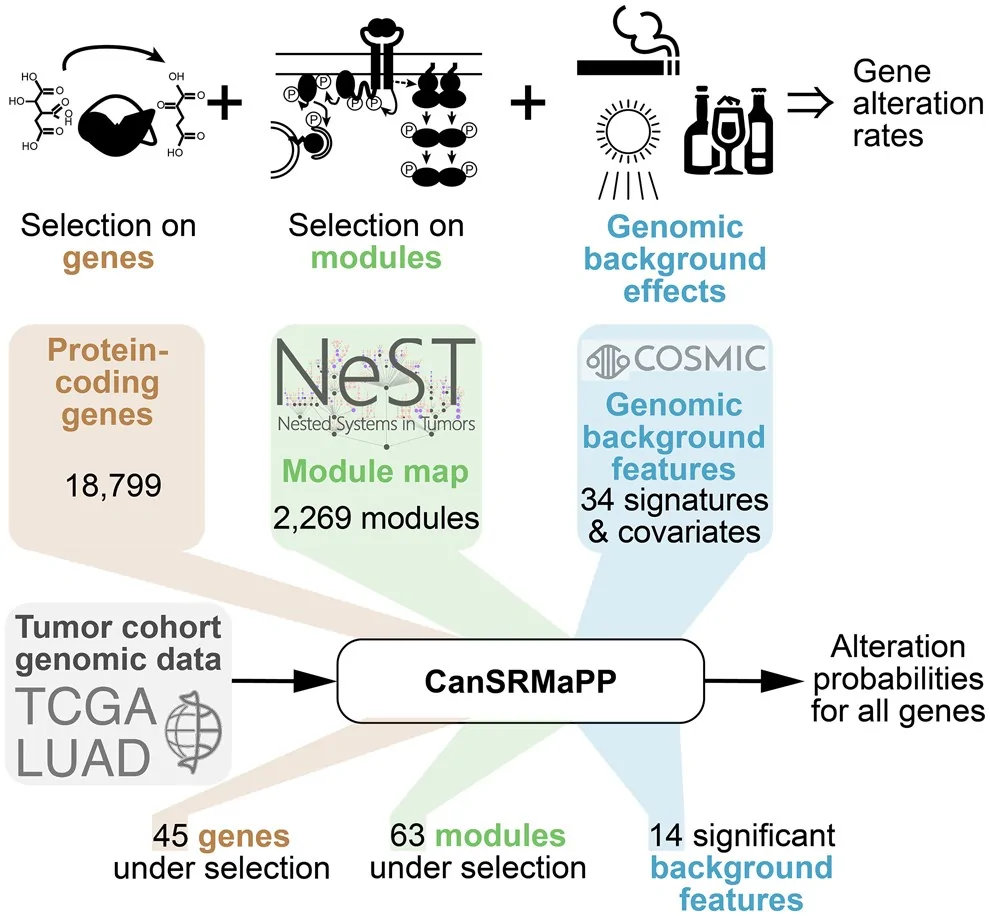
Interpreting tumor genomes through the CanSRMaPP model. Tumor genomic data are modeled to reveal selective pressures operating at the single-gene and multi-gene module levels, in addition to the underlying contributions of genomic mutational background processes. Inputs to CanSRMaPP supply a large feature space from which relatively few features are chosen, producing a parsimonious explanation of cohort-level gene alteration frequency.

In the present study, CanSRMaPP advances these concepts along several key lines. First, it simultaneously models selective pressures and genomic background processes, while HiSig only models selective pressures. Second, CanSRMaPP models four major classes of genetic alteration simultaneously (point mutations, copy number gain and losses, gene fusions), rather than a single type only. Third, CanSRMaPP more precisely discriminates among modules to select a parsimonious set contributing maximal information (Fig. 1). Using lung adenocarcinoma tumors and our previously developed cancer module map, NeST (Nested Systems in Tumors; Zheng *et al*. 2021), we identify positive selection on 63 multigenic modules, yielding a model which is highly predictive of mutation patterns in new lung cancer cohorts. These modules aggregate alterations across 18 811 genes, substantially expanding the collection of genes implicated in this cancer type. We further show CanSRMaPP’s adaptability to other tumor types and other module maps, presenting a general strategy for identifying cancer pathways and biomarkers.

## 2 Methods

### 2.1 CanSRMaPP model overview

CanSRMaPP models somatic genetic alteration frequencies in a cohort of tumor genomes from a weighted combination of evolutionary selection pressures and genomic background effects, collectively referred to as “features.” The evolutionary selection terms cover separate contributions from positive selection at the level of the gene and at the level of each module containing that gene. To account for synteny, positive selection on copy gains, copy losses, or fusions is broadcast to neighboring genes on the chromosome. The genomic background terms include COSMIC mutational (single-base substitutions/double-base substitutions/indels; SBS/DBS/ID) and copy-number (CN) signatures (Alexandrov *et al*. 2020, Steele *et al*. 2022), as well as the maximum transcript length of each gene and its relative replication timing (Lawrence *et al*. 2013, Martincorena *et al*. 2017; Fig. 1, Table S1, available as supplementary data at *Bioinformatics* online). The feature weights are optimized using a Bayesian framework which computes the likelihood of the observed frequency data under the model and a data-independent prior probability which favors parsimonious models with fewer nonzero feature weights. The product of the likelihood and prior probability yields the posterior probability, which serves as a performance score rewarding goodness-of-fit and penalizing model complexity.

### 2.2 Tumor genomic data

**Figure 2:**
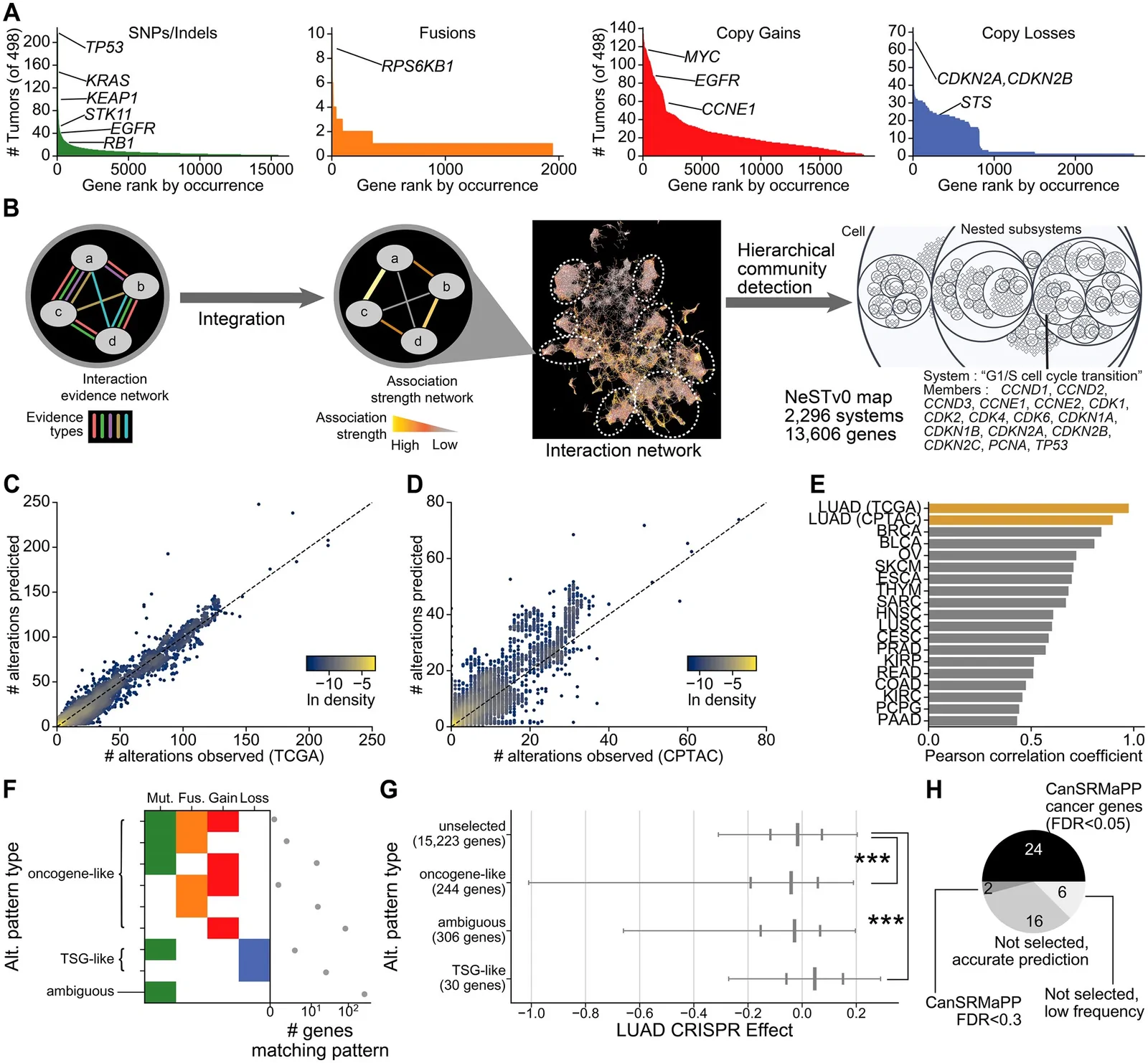
CanSRMaPP reconstructs LUAD alteration rates and recovers LUAD cancer genes. (A) Waterfall plots showing alteration event frequencies in the TCGA LUAD cohort. (B) Schematic describing construction of the NeSTv0 module map. (C) Scatter plot of observed alteration counts (*x* axis) vs. alteration counts predicted by CanSRMaPP (*y* axis) for the TCGA LUAD cohort (498 tumors). The colors at each point represent the local ln-density of observations. (D) Scatter plot of observed alteration counts (*x* axis) vs. alteration counts predicted by CanSRMaPP (*y* axis) for the CPTAC LUAD cohort (177 tumors). Colors as in A. (E) Pearson correlation coefficients of predicted vs expected values for the two LUAD cohorts (gold), as well as TCGA cohorts of other tumor types (gray). Abbreviations are standard OncoTree codes (Kundra *et al*. 2021). (F) LEFT: Heatmap indicating patterns of alteration under selection associated with different gene types. RIGHT: Number of genes under selection matching each alteration pattern. Alt.: alteration. (G) Plot of CRISPR knockout cell fitness phenotypes (*x* axis) in LUAD cell lines, as profiled in DepMap 24Q4, divided by alteration pattern type (*y* axis). Vertical lines indicate 5th, 25th, 50th, 75th, and 95th percentile from left to right. ***P < 10^−3^ by Mann-Whitney *U* test. The “unselected” category excludes “common essential” genes; note that not all genes are profiled by DepMap. (H) Pie chart of the 48 consensus cancer genes (recovered in ≥3 of 6 sources) categorized by their properties under CanSRMaPP.

CanSRMaPP takes as inputs frequencies of four types of genetic alteration for each gene: mutation (nucleotide substitution/indel), fusion, copy gain, and copy loss. We chose to develop CanSRMapp using the Cancer Genome Atlas (TCGA) lung adenocarcinoma (LUAD) cohort based on its sufficient size (498 tumors with data for analysis), high genetic diversity, and availability of data covering all four major alteration types across 18 811 protein-coding genes (The Cancer Genome Atlas Research Network 2014; Fig. 2A). The cohort had a mix of tobacco-smoking behaviors, with 40% of patients reporting no smoking history. Additionally, a separate cohort of 177 additional LUAD tumors from the Clinical Proteomic Tumor Analysis Consortium (CPTAC; Gillette *et al*. 2020) was analyzed for model validation.

All tumor genomics data were downloaded from the Genomic Data Commons [portal.gdc.cancer.gov] on 2 February 2024. Where available, the following data types were downloaded: “Gene Level Copy Number,” “Masked Somatic Mutation,” “Transcript Fusion,” “Allele-specific Copy Number Segment,” and “Annotated Somatic Mutation.” Protein-coding genes with the following properties were selected for analysis: (a) assigned to an NCBI identifier, EnsEMBL identifier and Human Gene Nomenclature Committee symbol(HGNC; Seal *et al*. 2023), (b) not a member of the HGNC olfactory receptor group (#141), and (c) expression (on a sample-by-sample basis) significantly more frequent than olfactory receptor genes by *χ*^2^ test. The use of olfactory receptors as a negative control follows previous work (Lawrence *et al*. 2013). Alteration events for each gene in each tumor were binarized at the gene level according to the following criteria. Copy-gain: at least four copies greater than normal tissue. Copy-loss: at least two copies fewer than normal tissue. Mutation: a gene was reported by a majority of somatic mutation callers to have at least one event in the following categories: “NMD_transcript_variant,” “missense_variant,” “splice_acceptor_variant,” “non_coding_transcript_exon_variant,” “splice_donor_variant,” “splice_region_variant,” “stop_gained,” “5_prime_UTR_variant,” “3_prime_UTR_variant.” Fusion: when reported by both the “STAR_Fusion” and “Arriba” transcript fusion pipelines. Only tumors measured for all four events were considered for analysis. While tumor-level binarization of each alteration obscures some information, the inference of cohort-level probabilities requires that the occurrence of each alteration be modelled as a Bernoulli trial.

### 2.3 Model architecture

The CanSRMaPP model follows a standard Bayesian framework, identifying the maximum *a posteriori* estimate of the model parameters w, v, z, E, c (see below) given the data (alteration frequencies from a tumor cohort under study):


(1) P(Model | Data)∝P(Data|Model) P(Model)

*P*(Data|Model), is defined as the likelihood of observing $k_{\tau ,g}$ occurrences of each alteration type τ∈ {mutation, fusion, gain, loss} for each gene g∈G in a cohort of tumors t∈T:


(2) $$P(\text{Data}|\text{Model})=\prod_{\tau }\;\prod_{g}\;\frac{\left|T\right|}{k_{\tau ,g}}p_{\tau ,g}{\;}^{k_{\tau ,g}}{\left(1-p_{\tau ,g}\right)}^{\left(|T|-k_{\tau ,g}\right)}$$

where p_*τ,g*_ is the probability that an alteration of type τ occurs in g. These probabilities are computed from the collection of selective and background features acting on each alteration, according to the logistic function:


(3) $$\log\frac{P}{1-P}=(R\times_{2}(E⊙((I\cdot w+H\cdot v{)}^{T}⊗1_{4})+J\cdot z+c⊗1_{\left|G\right|})$$

where the operator ×_2_ signifies batch matrix multiplication along the second axis of the operands and

**I** (|G|×|G|) is the identity matrix of rank |G|
**w** is a vector of length |G| containing selection weights on genes.
**H** (|G|×|M|) is a precomputed matrix that maps sets of genes to the modules m∈M that those genes comprise. **H** is defined with elements $h_{g,m}:=1$ for g∈m, else 0, after which each element is divided by the standard deviation of its column m, then rescaled so that the maximum value of **H** at any cell is 1.
**v** is a vector of length |M| containing selection weights on modules (optimized parameter).
**J** (4×|G*|*×|*B|*) is a precomputed tensor describing the expected influence of b∈B genomic background features on each of the 4 types of alterations to each gene *g*; see next section.
**z** is a vector of length |B| describing the overall importance of each background feature.
**c** is a vector of length 4 containing basal intercept values for the 4 alteration types.
**E** (4×|G|) describes how selection pressure on each gene is distributed across the four alteration types.
**R** (4×|G|×|G|) is a precomputed tensor capturing the effects of synteny; see below.

We note that under this model all alterations are conditionally independent given any shared influences from selection and genomic background. This simplifying assumption is necessary to avoid excessive computational complexity, though several violations of this assumption (i.e. mutual exclusivity or co-occurence of alterations) have been observed and/or proposed. A diagram of the matrix operations is shown in Fig. S1A, available as supplementary data at *Bioinformatics* online.

The second right-hand term in Equation (1), the model prior, is computed as:


(4) P(Model)=P(w)P(v)P(z)P(E)

Prior probabilities for w, v, and z are drawn from exponential distributions with hyperparameters $\lambda_{w}$, $\lambda_{v}$, and $\lambda_{z}$, respectively; the exponential distribution was chosen as the maximum-entropy distribution for nonnegative real values. Because gene-level selection pressures are equivalent to module-level pressures on single-member modules, the first two parameters were held equal ($\lambda_{w}{:=\lambda }_{v}$) for all analyses. E is drawn from |G| Dirichlet distributions, all with *α* as the concentration parameter for each alteration type; a Dirichlet distribution was chosen as the maximum-entropy distribution under the constraint that partition values sum to 1. Greater values of these hyperparameters serve to reduce the total number of features under selection (model regularization). The relationships between all variables are diagrammed in Fig. S1B, available as supplementary data at *Bioinformatics* online.

### 2.4 Module maps

For our primary analysis using CanSRMaPP, we used the NeSTv0 map from our previously published work, which defines modules as collections of genes (Zheng *et al*. 2021; Fig. 2B). Other module maps for analysis by CanSRMaPP were downloaded from the Gene Ontology (Ashburner *et al*. 2000), Reactome (Jassal *et al*. 2020), and CORUM (Tsitsiridis *et al*. 2023). Gene: module memberships were quantified as binary relationships. While many of the pathway map sources are hierarchical, map architecture requires only that (1) no modules are duplicated and (2) all memberships are binary. Modules with fewer than four or more than 256 members were discarded, so that modules conveyed meaningfully different information from single genes while remaining interpretable (Zheng *et al*. 2021).

### 2.5 Precomputed model elements

The influence of each genomic background feature on each genetic alteration is precomputed in tensor **J** with dimensionality 4×|*G|*×|*B|*. A total of 134 genomic background features b∈B are considered, including 34 published mutational signatures 151 651 of which 67 are related to Single-Base Substitutions (SBS), 19 to Double-Base Substitutions (DBS), 23 to Insertions/Deletions (ID) and 25 to copy-number aberrations (CN). Each signature is associated with a vector of activities across tumors, $a_{b}=(a_{b,1},a_{b,2},\ldots ,a_{b,|T|})$ calculated from the tumor genomic data using the tool SigProfilerAssignment (Díaz-Gay *et al*. 2023). As a first step, signatures presenting activity > 0 in fewer than 5% of patients are discarded; for LUAD, 32 signatures remained under consideration (13 SBS, 2 ID, 5 DBS, 12 CN), with different sets of signatures potentially carrying relevance for different tumor types (Table S1, available as supplementary data at *Bioinformatics* online) Activity values for each b are normalized to the range [0,1] by linear scaling. Meanwhile, the binarized occurrence (see above) of a particular genetic alteration (type τ, gene g) across tumors is represented as the observation vector $o_{\tau ,g}=(o_{\tau ,g,1},o_{\tau ,g,2},\ldots ,o_{\tau ,g,|T|})$. For these mutational signature features, elements of **J**, the background influence tensor, are defined using the Pearson correlation:


(5) $$j_{\tau ,g,b}=Pearson(a_{b}{,o}_{\tau ,g})$$

Two further genomic background features are ORF length and replication timing. For the background feature relating to ORF length:


(6) $$j_{\tau =\mathrm{mutation},g,b=\mathrm{ORF}\;\mathrm{length}}=\mathrm{length}(g)$$

where length denotes the number of nucleotides in the longest RefSeq (release 226) transcript of that gene, normalized to the [0,1] range by linear scaling. For the background feature relating to replication timing:


(7) $${j_{\tau =\mathrm{mutation},g,b=\mathrm{timing}}}^{=}\mathrm{SigProfilerTopograph}(g)$$

SigProfilerTopography (Otlu and Alexandrov 2024) is a published tool used to score genes by their relative replication timing; values are normalized to the [0,1] range by linear scaling.

Elements of **R**, the synteny broadcast tensor, propagate selection on frequent fusion, copy gain, and copy loss alterations τ to neighboring genes (g,f) on the same chromosome arm c. Employing observations **o** as described above, values of $R_{\tau ,g,f}$ are calculated as Pearson correlations:


(8) $$r_{\tau ,g,f}\;=\;\{{\;}_{0\;\mathrm{otherwise}}^{\in \{fusion,\;gain,\;loss\}\;\cap \;g\;\in \;F_{\tau }\;\cap \;c(g)\;=\;c(f):\;Pearson(o_{\tau ,g},\;o_{\tau ,f})}\}$$

$F_{\tau }$ denotes the set of genes in the top 20% most frequently affected by alteration type. This restriction was chosen to reduce computational complexity (Supp. Note 2, available as supplementary data at *Bioinformatics* online). For copy-number alterations, $F_{\tau }$ was further restricted to the 5% of genes most frequently altered on that chromosome and arm. Values of **J** or R which are negative or for which the correlation is not significant (*P *> 10^−3^ by Student’s *T*-test) are set to 0.

### 2.6 Model implementation

CanSRMaPP was coded in Python using PyTorch (Ansel *et al*. 2024) and its parameters **w**, **v**, **z**, and **E** optimized using the Adam optimizer, which was chosen for its observed greater speed and consistency in identifying maximum-posterior solutions vs. other optimizers, including those based on Monte Carlo Markov Chains (MCMC; Supp. Note 2, available as supplementary data at *Bioinformatics* online). In each CanSRMaPP run, the learning rate is initialized to 10^−2^, cycling using cosine annealing with warm restarts(Loshchilov and Hutter 2016). For 3000 epochs, the gradient is perturbed with random noise ∼N(0,0.01). For the next 3000 epochs, the noise perturbation is removed and the model parameters and posterior probability are inspected each epoch. Weights are initialized equal to 1 for six independent chains, which are then reinitialized 9 times (for a total of 10 runs) based on the outcomes of previous optimization runs. Model parameters with the greatest posterior probability across all chains and runs were stored.

### 2.7 Identification of alterations under selection

To identify alterations (type τ, gene g) under selection, an alternative probability **Q** was calculated


(9) $$\log\frac{Q}{1-Q}=\log\frac{P}{1-P}-(E⊙((I\cdot w+H\cdot v{)}^{T}⊗1_{4})$$

effectively ablating non-syntenic influences of selection on predicted alteration frequencies. For each alteration, the probability of its observation is given by the probability mass function of the binomial distribution:


(10) $$P(k_{\tau ,g}=K|p_{\tau ,g})=\frac{\left|T\right|}{K}{p_{\tau ,g}}^{K}(1-p_{\tau ,g}{)}^{\left(|T|-k\right)}$$

Then a likelihood ratio $L_{\tau ,g}={P(k_{\tau ,g}|p_{\tau ,g})/P(k_{\tau ,g}|q}_{\tau ,g})$ was calculated for each observation. A *P-*value was calculated as the probability over all observation counts that $L_{\tau ,g}$ would be exceeded by chance in a cohort simulated under the ablated model:


(11) $${p‐value}_{\tau ,g}=\sum_{x=0}^{\left|T\right|}\;P(k_{\tau ,g}=x|q_{\tau ,g})\cdot 1[\frac{P(x|p_{\tau ,g})}{{P(x|q}_{\tau ,g})}>\frac{P(k_{\tau ,g}|p_{\tau ,g})}{{P(k_{\tau ,g}|q}_{\tau ,g})}]$$

We then computed a false discovery rate using the Benjamini-Hochberg method; alterations with FDR < 0.05 were considered under selection.

### 2.8 Hyperparameter optimization

We reasoned that, with optimal hyperparameters, model posterior probability should be significantly greater when supplied with a biologically relevant module map (NeSTv0; H in Equation 3) than when supplied with an empty (null) module map, but that spurious module maps should not show such advantages. To generate randomized maps for controls whose structure mimicked NeSTv0, modules m were repopulated by selecting |m| genes g without replacement, where the probability of selecting a given g was proportional to the number of NeSTv0 modules containing g:


(12) $$P(g)\propto \;\sum_{m\in M}^{\;}\;1(g\in m)$$

The collection of available genes was re-initialized after populating each module. This weighting preserves the frequency of gene memberships. To allow comparison of differently-sized models (i.e. from different module maps) after optimization, a corrected posterior was calculated in which P(v) was computed only for nonzero elements of v. This corrected posterior is used in all plots and tables. We tested values of the hyperparameters *λ*_wv_, *λ_z_*, and *α* from 1.0 to 4.0, using 3 chains with 4 cycles each. We then determined a set of Pareto-optimal hyperparameter values *λ_**w**_*, *λ_**z**_*, and α with respect to (a) maximal difference between the NeSTv0 and null models and (b) minimal difference between the randomized and null models (Fig. S2C, Table S2, available as supplementary data at *Bioinformatics* online). We found that *λ_z_* generally showed minimal influence on model composition or performance (not shown).

For a second means of determining hyperparameter suitability, we employed the curated OncoKB knowledgebase (Chakravarty *et al*. 2017) to obtain a list of all gene-level alterations of all four types annotated as “Oncogenic.” We compared this list with the alterations predicted to be under selection (see above) by each NeSTv0 model to obtain precision and recall measurements, defining a second set of pareto-optimal hyperparameter values with respect to precision and recall (Fig. S2D, Table S2, available as supplementary data at *Bioinformatics* online). Hyperparameters were chosen from among the intersection of the two pareto-optimal sets. Selected hyperparameter values *λ*_wv_ = 2.75, *λ_z_* = 1.0, and *α* = 2.50.

### 2.9 Comparison of module maps

To assess similarity of module maps (after cancer association had been assessed, either through use of CanSRMaPP or as a reported result of some other publication) the following similarity score was calculated:


(13) $$\frac{\sum_{m_{1}\in M_{1}}^{\;}\;match(m_{1},M_{2})+\sum_{m_{2}\in M_{2}}^{\;}\;match(m_{2},{M_{1})}_{\;}}{\left|M_{1}|+|M_{2}\right|}$$

where $m_{1}$ and $m_{2}$ are members of module maps $M_{1}$ and $M_{2}$ respectively, and where match(m′,M”) = 1 when the Jaccard similarity between m′ and any m” ∈M” exceeds 0.5 and 0 otherwise. Thus, the score is the fraction of modules across both maps for which a similar module can be found in the other map.

### 2.10 Using CanSRMaPP features to predict protein readouts

Reverse-phase protein array (RPPA) data for the TCGA-LUAD samples were downloaded from portal.gdc.cancer.gov on 26 June 2024. Briefly, RPPA uses antibody probes to measure abundance unmodified or posttranslationally modified proteins in each tumor. We refer to such measurements as protein “readouts.” For each readout, tumors lacking either genomic data or an RPPA measurement were discarded, producing variable *n* for each readout. RPPA measurements were normalized such that the interquartile range to [–1,1] with the median value at 0. Readouts were modeled as functions of genomic alterations for the purposes of linking genetic alterations to “molecular phenotypes” defined by protein readouts. Genomic alterations were featurized in terms of gene panels or in terms of significant features from CanSRMaPP output. For a gene panel G, we represented the profiled cohort as **X**, where for each tumor t∈T and gene g∈G, where G⊆G. $X_{i,g}:=$1 if any alteration in g was present in i and 0 otherwise. If a gene was more frequently the subject of copy gain than deletion, its copy losses were not considered, and *vice versa*, since it is implausible that both copy gain and loss of a single gene are under selection in the same tumor type. When using CanSRMaPP features, genes were considered altered if any alteration type under selection was observed in that tumor; modules were considered altered if any of their member genes were altered by the same criteria. Signature activities of significant genomic background processes were normalized to the [0,1] range; discarding “length” and “timing” since observations were tumors, and not genes. For each RPPA readout, linear models were fit using the ElasticNet loss function $L=\frac{1}{2n}||y-Xw|{|}^{2}+\lambda_{2}||w|{|}^{2}+\lambda_{1}||w||$, where **y** represents the RPPA signals, and w are the feature weights. Models were fit and assessed by nested five-fold cross validation; the hyperparameters $\lambda_{1}$ and $\lambda_{2}$ were optimized within the training split for each readout/model prior to assessment on evaluation data. For each RPPA probe, models were scored by the Spearman correlation (to accommodate assay noise) between predicted and observed values across an unseen test set of 20% of tumors. Models with positive significant correlation were considered successful (*P* < 0.05). Gene panel sources are as follows. TCGA LUAD drivers: ref. (The Cancer Genome Atlas Research Network 2014) COSMICv100: COSMIC v100 release, “LUAD” for genes driving lung adenocarcinoma; “Ep. & other”: tissue types “E” and “O.” All other gene panels are sourced from OncoKB (Chakravarty *et al*. 2017).

## 3 Results

### 3.1 CanSRMaPP captures genome-wide lung adenocarcinoma alteration rates

We first optimized model parameters as described using the TCGA lung adenocarcinoma (LUAD) tumor cohort, producing a model of the LUAD genome (Fig. 2A, *Methods*). For definitions of gene modules, we used our previously developed cancer module map, NeST (Zheng *et al*. 2021; Nested Systems in Tumors), based on hierarchical clustering of a protein interaction network derived from proteomic characterizations of cancer cells (Fig. 2B).

Despite the simplified representation of the genome present in the model, we found input alteration frequencies to be recovered with high accuracy (Pearson’s *r *= 0.98; Fig. 2C). Copy gains and losses were modeled best (*r *= 0.97 and 0.96, respectively) with somewhat poorer performance for substitutions and indels (*r *= 0.86) and gene fusions (*r *= 0.53) which occur more rarely and less predictably (Fig. S2A, available as supplementary data at *Bioinformatics* online). To test whether the CanSRMaPP cancer genome model could translate to an independent validation cohort without further optimization, we obtained genomic data for 177 additional LUAD tumors from the Clinical Proteomic Tumor Analysis Consortium (CPTAC; Gillette *et al*. 2020). We found that the alteration frequencies expected by the CanSRMaPP model were well-matched to those observed in CPTAC (*r *= 0.90), although a systematic tendency toward higher-than-expected copy gain and loss frequency was observed, possibly due to differences in computational processing (Figs. 2D, S2B, available as supplementary data at *Bioinformatics* online). By comparison, predictions on other tissue types correlated more poorly (Fig. 2E), indicating that CanSRMaPP captures a degree of disease specificity.

The CanSRMaPP model architecture permits the decomposition of features contributing to each gene-level alteration such that selection pressures can be separated from influences of non-selective factors (e.g. tobacco smoking). Of the 18 811 genes included in the model, 422 showed evidence—after accounting for genomic background features—of at least one alteration type under selection with FDR < 0.05 (Tables S3 and S4, Methods, available as supplementary data at *Bioinformatics* online) and were accordingly denoted “CanSRMaPP LUAD genes.” To corroborate the involvement of these genes in cancer progression, we analyzed CRISPR gene knockout effects among LUAD cell lines profiled by the DepMap resource (Meyers *et al*. 2017, Tsherniak *et al*. 2017, DepMap B 2024). Each CanSRMaPP cancer gene was assigned an alteration pattern type: “oncogene-like” if under selection by copy gain or fusion (with or without selection on mutations), “tumor suppressor gene (TSG)-like” if by copy loss (with or without selection on mutations), or “ambiguous” if solely under selection by mutation (Fig. 2F). We found that knockouts of oncogene-like genes had significantly more detrimental, and TSG-like genes more salutary, effects in LUAD cell lines (Fig. 2G, *P *< 10^−3^ by Mann-Whitney *U* test for both comparisons). To assess the degree to which CanSRMaPP agreed with other genomic analyses, we assembled six previous studies by TCGA and others examining the same LUAD cohort (The Cancer Genome Atlas Research Network 2014, Leiserson *et al*. 2015, Bailey *et al*. 2018, Repana *et al*. 2019, Zheng *et al*. 2021, Sondka *et al*. 2024). Though these studies diverged from one another (Fig. S2C, available as supplementary data at *Bioinformatics* online), we identified a set of 48 “consensus cancer genes” identified in at least three prior analyses. Of these 48 genes, 24 were among those implicated as LUAD genes by CanSRMaPP (Fig. 2H). The remaining consensus genes were either low-confidence hits (2 genes, FDR < 0.3), well-explained by CanSRMaPP using genomic background features (16 genes) or were rarely altered (6 genes in < 4% of tumors, Fig. S2D, available as supplementary data at *Bioinformatics* online).

### 3.2 Expanding the landscape of lung cancer avlterations via multigene modules

**Figure 3:**
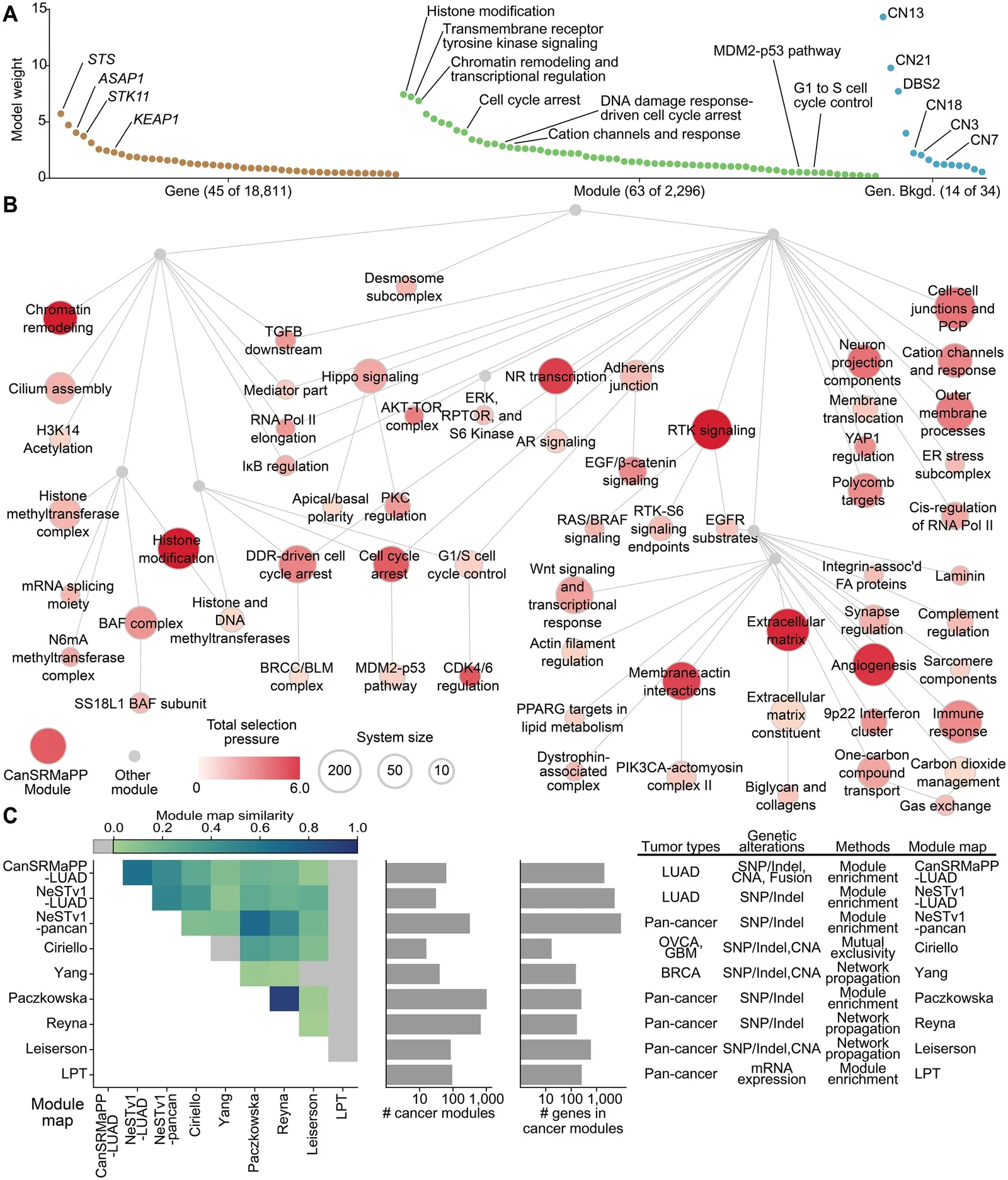
A limited set of features describes patterns of somatic mutation in LUAD. (A) Weights assigned to genes, NeST modules, and mutational signatures; features with 0 weight not shown. (B) Network diagram mapping CanSRMaPP modules. Red shading of nodes indicates total selection pressure (unitless). Node sizes are scaled to the logarithm of the total number of genes. “AR”, androgen receptor; “BAF”, BRG1/BRM-associated feature complex; “DDR”, DNA damage response; “N6mA”, N6-methyladenosine; “ER”, endoplasmic reticulum, N6-methyl adenosine; “PCP”, planar cell polarity; “Pol”, polymerase; “PKC”, protein kinase C; “S6”, ribosomal protein S6. Other acronyms are official gene and/or protein symbols. Additional nodes from the NeSTv0 hierarchy are included to link the CanSRMaPP modules. (C) Comparison of cancer module maps. From left to right: Heatmap showing inter-map similarity, defined as the fraction of modules from both maps that can be recovered in the other (*Methods*); number of cancer modules in each map; number of genes mapped to those modules; comparison of the provenance of each map.

Inspection of the model identified influences from 63 module-level and 45 gene-level selective pressure features, and from 14 genomic background features (total 114 features; Fig. 3A and B). The highest-weighted features were the copy-number signature (CN) genomic background feature “CN13,” associated with loss of heterozygosity, (Steele *et al*. 2022) followed by two modules, “Histone modification” (29 genes including TP53, CREBBP and RB1) and “RTK signaling” (27 genes including KRAS, EGFR and TP53). Of the 422 CanSRMaPP LUAD genes, 389 were included in at least one of the modules under selection. We next compared cancer module maps from prior publications(Ciriello *et al*. 2013a, Leiserson *et al*. 2015, Paczkowska *et al*. 2020, Reyna *et al*. 2020, Yang *et al*. 2021, Li *et al*. 2023). We found that nearly all such sources diverged greatly with one another, likely due to differences not only in the methodology of module detection, but the cohorts under examination and the nature of the input data (Fig. 3C, Supplementary Methods, available as supplementary data at *Bioinformatics* online).

**Figure 4:**
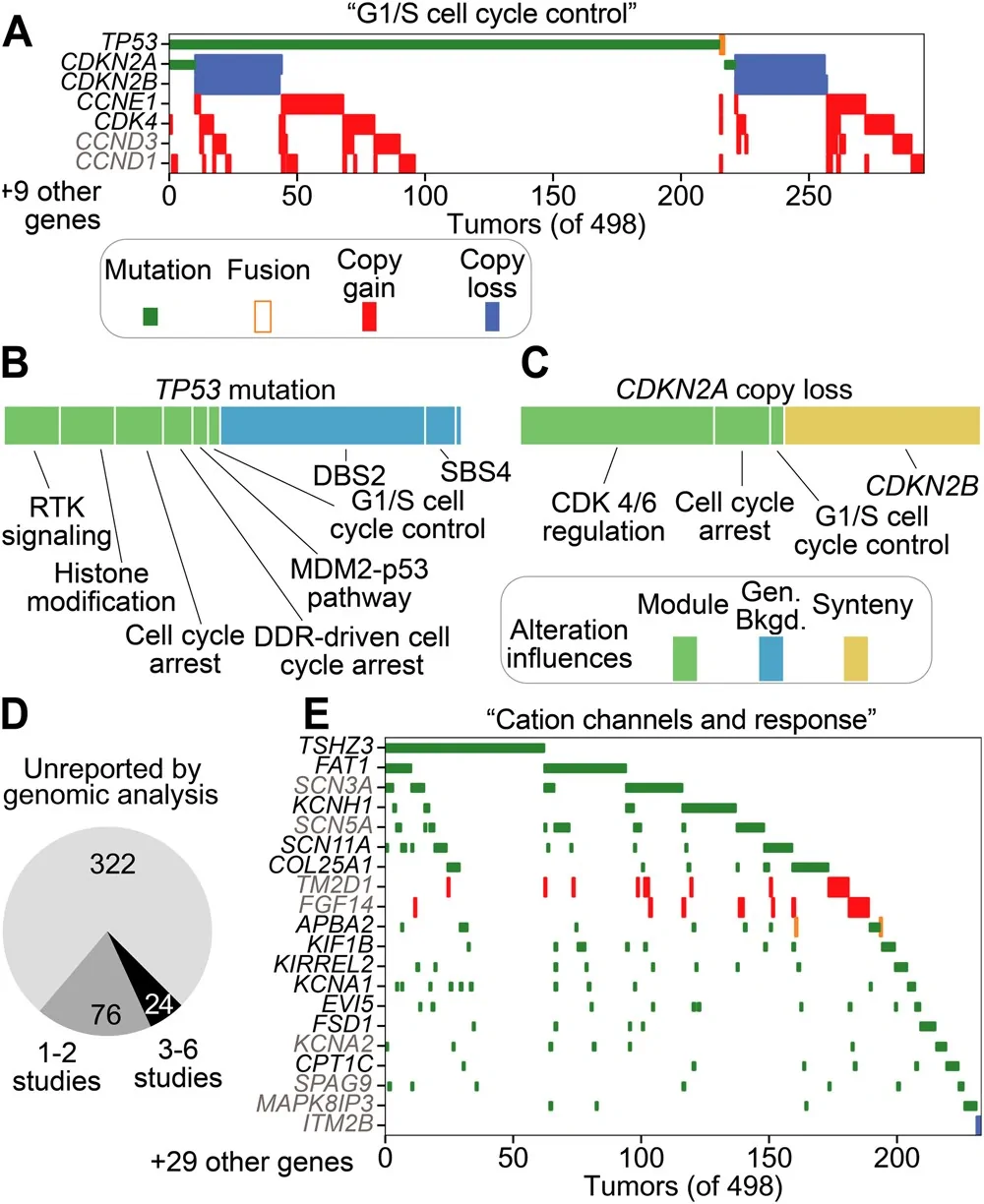
Multigene modules describe selection pressures on LUAD cancer genes. (A) Oncoprint showing patterns of selected genetic alterations in genes of the “G1/S cell cycle control” module (rows) by lung cancer patients (columns; TCGA LUAD cohort). Colors represent mutation (green), fusion (hollow red), copy gain (solid red), or copy loss (blue). Genes with faded symbols are under selection with low confidence (FDR < 0.3 vs. FDR < 0.05 for others). Only genes with at least one alteration under selection are shown (7/16); only tumors with at least one alteration in those genes are shown (295/498). Genes are sorted in descending order of alteration frequency; tumors are sorted by alteration state of genes following the gene order. (B, C) Diagrams of relative contributions of feature weights to TP53 mutation and CDKN2A copy loss respectively. Green, blue and yellow regions denote features related to modules, background, or synteny, respectively (see legend at right). (D) Pie chart describing the number of other sources nominating CanSRMaPP-selected genes. (E) Oncoprint of CanSRMaPP genes under selection in the “Cation channels and response” module. Display as for panel A, with 233/498 tumors shown and 20/49 genes shown.

Many prominent oncogenes and tumor suppressors were not assigned a single-gene weight, but instead organized into one or more modules. For example, the “G1/S cell cycle control” module contained 7 genes under selection (FDR < 0.05) in LUAD (2 with FDR < 0.3, 16 genes total in module), combining *TP53* with prominent cyclins, cyclin-dependent kinases, and CDK inhibitors (Fig. 4A). Moreover, six of those genes received selective pressure from multiple modules. For example, mutation of the tumor suppressor *TP53* (tumor protein p53) was assigned selective pressure from memberships in a total of four modules (Fig. 4B), and copy loss of *CDKN2A* (Fig. 4C) was additionally explained by selective pressure on “CDK4/6 regulation” and “Cell cycle arrest,” as well as synteny with *CDKN2B*.

We now turned to the majority of genes implicated by CanSRMaPP which had not been previously reported in any of the six previous LUAD genomics studies (322/422; Fig. 4D). Many of these genes were attributed to four modules in particular, “Extracellular matrix” (30 new genes), “Endothelial Cell Function and Angiogenesis” (29), “Histone modification (22),” and “Receptor tyrosine kinase signaling” (15), all of which relate to hallmark processes of LUAD tumor biology (Cooper *et al*. 2013, Pickup *et al*. 2014, Audia and Campbell 2016, De Palma and Hanahan 2024). Other novel cancer genes were from the “Cell-cell junctions and Planar Cell Polarity” module (22), related to multifaceted processes that are disrupted, rather than co-opted, by tumorigenesis. We also noted unexpected groupings of new cancer genes. In “Cation channels and response” (Fig. 4E), only one CanSRMaPP cancer gene, *FAT1*, had been nominated by previous omics studies (Bailey *et al*. 2018, Repana *et al*. 2019); the remaining 11 high-confidence (FDR < 0.05) and 8 lower-confidence (FDR < 0.3) genes in this module had not. Notably, the genes in this module encoding voltage-gated ion channels are under investigation as therapeutic targets (e.g. *SCN3A;*  Angus and Ruben 2019, Zúñiga *et al*. 2022), and the most frequently altered gene in this module, *TSHZ3*, is a known suppressor of the pro-apoptotic protein CASP4 (Kajiwara *et al*. 2009).

### 3.3 CanSRMaPP extends to multiple module maps and tumor types

As an initial demonstration of the flexibility of CanSRMaPP, we fit new models using other module maps of cell biology: the Gene Ontology (GO; Ashburner *et al*. 2000), Reactome (Jassal *et al*. 2020), and CORUM (Tsitsiridis *et al*. 2023; Fig. S3, Tables S3 and S4, available as supplementary data at *Bioinformatics* online). All four models produced solutions with Pearson correlations comparable to NeST (Fig. S3A, available as supplementary data at *Bioinformatics* online), and all models except that using CORUM had significantly greater posterior probabilities than equivalent randomized maps (Fig. S3B, available as supplementary data at *Bioinformatics* online). The models using NeSTv0 (i.e. CanSRMaPP-LUAD) and Reactome included nearly identical numbers of features of each type, while GO included far more, and CORUM far fewer, systems (Fig. S3C, available as supplementary data at *Bioinformatics* online). Despite these different compositions, the modules recovered similar module sets and considered similar sets of genes to be under selection, with CORUM showing some divergence from the other three (Fig. S3D–F, available as supplementary data at *Bioinformatics* online).

We also applied CanSRMaPP, using the NeSTv0 systems map, to 19 other TCGA tumor cohorts (Fig. S4A–C, Tables S3 and S4, available as supplementary data at *Bioinformatics* online). The resulting systems models included varying numbers of features, with the 122 features in CanSRMaPP-LUAD representing an intermediate value, and the model of breast cancer (BRCA) presenting a notable outlier with over 1000 features included. We found a notable, but not deterministic, relationship between the number of alteration events per tumor and the number of features included in the resulting model (Pearson r = 0.62, Fig. S4D, available as supplementary data at *Bioinformatics* online).

### 3.4 CanSRMaPP provides a basis for predicting cancer molecular phenotypes

**Figure 5:**
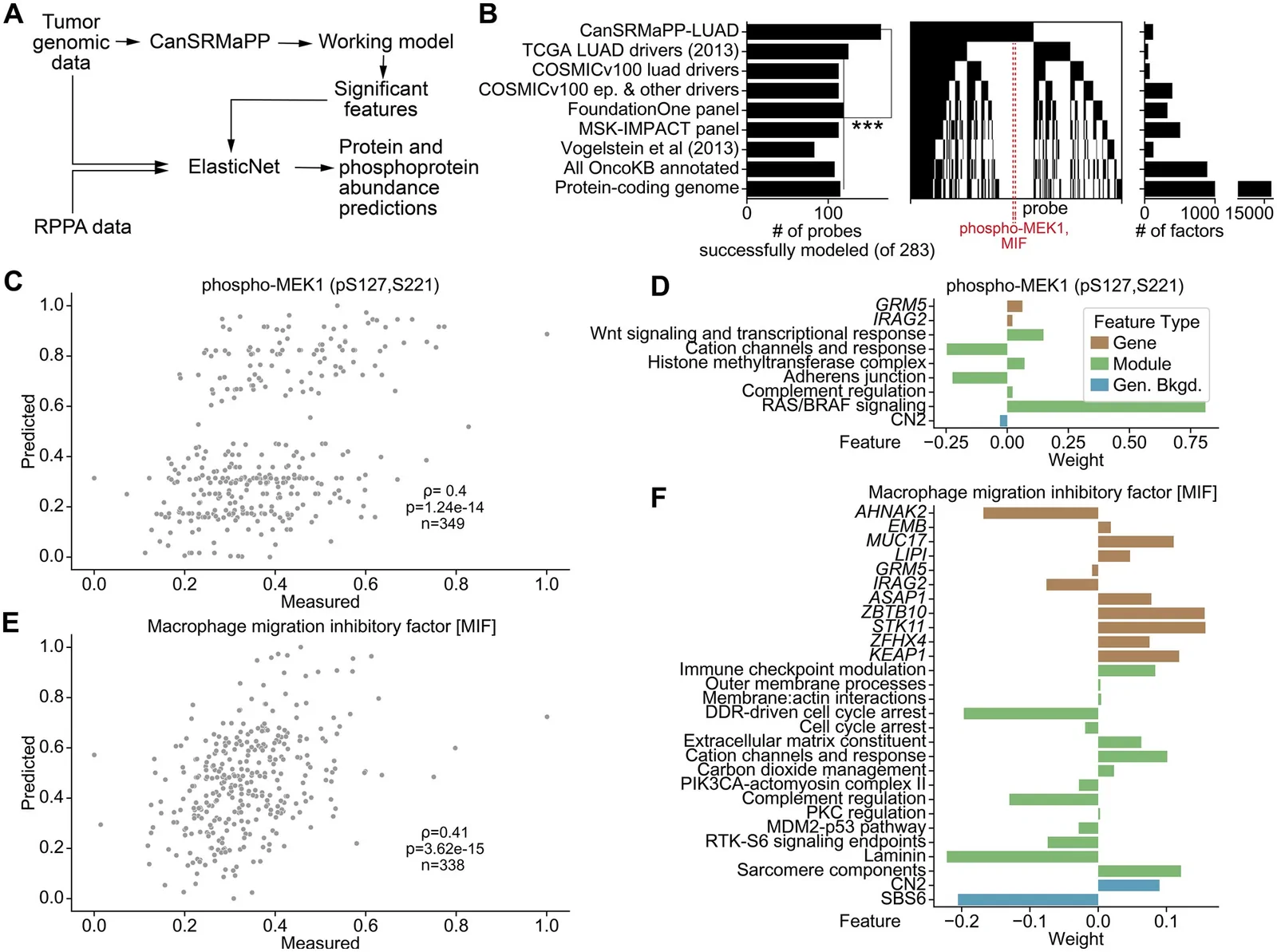
CanSRMaPP features enable discovery of links between genotype and molecular phenotype. (A) CanSRMaPP features are used to explain protein and phospho-protein levels measured by reverse phase protein arrays (RPPA). Each tumor is described in terms of its profile of alterations to CanSRMaPP features. (B) LEFT: Number of readouts successfully modeled using features from the indicated sources. RIGHT: Heatmap showing successfully (black) and unsuccessfully (white) modeled readouts (*x* axis), grouped by the source of model input features (*y* axis). Solid red line marks the set of 50 readouts uniquely modeled by CanSRMaPP; the dashed line indicates the positions of phospho-MEK1 (MEK1-pS217S221) and total MIF (Macrophage Migration Inhibitory Factor) readouts. The far right panel shows the number of features covered by each source of features. “Ep.”, epithelial. ***P < 0.001 by McNemar’s Test. All reference gene panels derive from OncoKB (Chakravarty *et al*. 2017). (C) Scatter plot of measured levels vs. levels predicted from CanSRMaPP features for phospho-MEK1. Levels are normalized on both axes to the [0,1] range. *ρ*, Spearman correlation; Gen. Bkgd., genomic background. (D) Features contributing to the model of phospho-MEK1 levels. (E) Plot of measured vs. predicted MIF levels as in C. (F) Features contributing to the model of MIF levels.

We next sought to use the key genes, modules and background features identified by CanSRMaPP to model lung tumor molecular phenotypes as an indication of how it might inform other tumor modeling tasks (Park *et al*. 2024, Zhao *et al*. 2024). To cover a wide phenotypic range, we examined data from 468 reverse-phase protein array (RPPA) experiments conducted on the TCGA LUAD cohort. RPPA experiments use antibody probes to produce readouts of key cancer protein abundances in unmodified and posttranslationally modified isoforms. To predict these protein levels, we represented each tumor by its profile of alterations to CanSRMaPP features (Fig. 3A, *Methods*), which was then used to predict each RPPA protein level via regularized linear regression (Friedman *et al*. 2010). Models were considered successful when predicted levels for an unseen test correlated positively and significantly with measured protein levels, and unsuccessful otherwise (Fig. 5A, *Methods*). Overall, 177 protein readouts could be successfully modeled with the CanSRMaPP feature profile. For reference, we compared these results to analogous regression models based on gene-level features. The models using CanSRMaPP features outperformed all others, and for 46 protein levels, successful models were only computed using CanSRMaPP features (*P *< 10^−3^ by McNemar’s test; Figs. 5B, S5, Table S5, available as supplementary data at *Bioinformatics* online).

The resulting CanSRMaPP-based models captured known protein regulatory relationships. For example, phospho-MEK1 levels were most strongly influenced by alterations in the “RAS/BRAF” module, which contains its known upstream regulators (Fig. 5C, D). Another example was abundance of Macrophage migration inhibitory factor (MIF), which is a secreted factor that assists tumor immune evasion (Balogh *et al*. 2018). Overall MIF levels were strongly influenced by genetic alterations in modules influencing immune response and evasion such as “Laminin,” “Complement regulation,” and “Immune checkpoint modulation” (Fig. 5E, F). Notably, both examples were among the 46 readouts modeled using CanSRMaPP features and not using any purely gene-based model.

## 4 Discussion

It has long been noted that alterations to different genes in cancer can disrupt the same pathways (Fearon and Vogelstein 1990). While this observation has guided cancer research for decades and helped expand the set of putative cancer-driving genes (Leiserson *et al*. 2015, Reyna *et al*. 2018, Kim *et al*. 2021, Swaney *et al*. 2021, Sondka *et al*. 2024), methodologies to define an ensemble of cancer modules for any given tumor type are still developing. Here, CanSRMaPP advances such methodology along several key lines. First, it simultaneously models selective pressures and genomic background processes without supervision. In contrast, current methods for cancer gene identification tend to treat driver alterations as outliers from a background model (Lawrence *et al*. 2013, Leiserson *et al*. 2015, Bailey *et al*. 2018, Reyna *et al*. 2018, Paczkowska *et al*. 2020, Reyna *et al*. 2020) or else rely on prior annotations of known cancer genes for trained classification (Bailey *et al*. 2018, Tokheim and Karchin 2019). Second, CanSRMaPP models four major classes of genetic alteration simultaneously, rather than relying on separate models for different types. Third, it explicitly discriminates among modules to select a parsimonious set contributing maximal information, rather than testing each module for enrichment individually. These advantages lead to a substantial expansion in the set of lung adenocarcinoma (LUAD) genes coupled with organisation of these genes into 63 distinct cancer modules (Fig. 3A and B).

Some potential limitations of CanSRMaPP are as follows. First, tumor genomic data with sufficient depth and breadth to train CanSRMaPP are only currently available from bulk samples; thus, CanSRMaPP does not attempt to detect polyclonality within a single tumor sample. Second, CanSRMaPP requires genome-wide sequencing data for training. Since gene panel sequencing is more common, applying CanSRMaPP to newly identified subtypes or rarer tumors will require dedicated clinical effort. Third, CanSRMaPP assumes that selection strength on a module is applied equally to all substituent genes. Microenvironmental context, paralogous genes, phenotypic penetrance of possible variants, and many other factors may play a role in modulating the influence of each specific alteration on the activity of the module in which it participates. Finally, CanSRMaPP does not attempt to model nonlinear relationships among combinations of genetic alterations, such as mutual exclusivity or co-occurrence (Babur *et al*. 2015, van de Haar *et al*. 2019).

Whatever the impact of these limitations, CanSRMaPP produced a working model that usefully reconstructs LUAD somatic alteration patterns across 498 tumors and 18 811 genes with only 122 distinct features (Figs 2C and 3A). The model accurately predicts alteration frequencies in an independent tumor cohort, demonstrating that this condensed representation of the LUAD genome preserves key information about the biology of the disease (Fig. 2D and E). Moreover, without further information, it forms a foundation for classifying alterations by their functional impact (Fig. 2F and G).

A key emerging application for module maps is in interpretable machine learning models, where multigenic modules represent functional units governing a particular molecular phenotype (Li *et al*. 2023, 2025, Kong *et al*. 2024, Park *et al*. 2024, Zhao *et al*. 2024). To assess the relevance of CanSRMaPP in predicting a broad spectrum of tumor molecular phenotypes, we used selected features as a basis for modeling RPPA readouts, which reflect a variety of signaling, DNA damage, growth, and other responses. In this exercise, the CanSRMaPP approach was uniquely able to translate the tumor genome to actionable information– e.g. prediction of phospho-MEK1 within a signaling pathway targeted by multiple drugs (The Cancer Genome Atlas Research Network 2014) and prediction of MIF1 levels which aid immune evasion (Balogh *et al*. 2018; Fig. 5C–F). Therefore, in addition to understanding patterns of genetic mutation, CanSRMaPP enables downstream explanation of phenotypic presentation.

Together, these results show that CanSRMaPP provides a strong and versatile foundation for more powerful tumor genomic modeling. We note that in our selection of hyperparameters we relied upon a curated database, OncoKB whose utility varies tumor type (Chakravarty *et al*. 2017; Fig. S1D, available as supplementary data at *Bioinformatics* online). We did not re-optimize hyperparameters for CanSRMaPP models of other tumors, which may be a beneficial step to take in some cases (Fig. S4, available as supplementary data at *Bioinformatics* online). However, CanSRMaPP was highly adaptable to the use of other module maps and could maintain consistency in predictions across different maps (Fig. S3, available as supplementary data at *Bioinformatics* online). Finally, this work demonstrates a more general framework for concurrent modeling of disease-linked and confounding influences on variant frequency that is, in principle, translatable to other polygenic diseases.

## Supplementary Material

btag668_Supplementary_Data

## Data Availability

The data underlying this article are available on Zenodo at https://doi.org/10.5281/zenodo.21879470. Software implementation of CanSRMaPP and associated analyses can be found at https://github.com/idekerlab/cansrmapp. Preprocessed tumor genomics data constitute a de-identified data derivative under the NIH universal Data Use Certification, are hosted at the above links. Source data can be found at gdc.cancer.gov subject to NIH restrictions on access.

## References

[btag668-B1] Alexandrov LB , Kim J, Haradhvala NJ et al The repertoire of mutational signatures in human cancer. Nature 2020;578:94–101. 10.1038/s41586-020-1943-3 PMC7054213 32025018

[btag668-B2] Angus M , Ruben P. Voltage gated sodium channels in cancer and their potential mechanisms of action. Channels (Austin) 2019;13:400–9. 10.1080/19336950.2019.1666455 PMC6768049 31510893

[btag668-B3] Ansel J , Yang E, He H et al PyTorch 2: faster machine learning through dynamic python bytecode transformation and graph compilation. In: *Proceedings of the 29th ACM International Conference on Architectural Support for Programming Languages and Operating Systems*, Volume 2. New York, NY: ACM; 2024.

[btag668-B4] Arnaud O , Le Loarer F, Tirode F. BAFfling pathologies: alterations of BAF complexes in cancer. Cancer Lett 2018;419:266–79. 10.1016/j.canlet.2018.01.046 29374542

[btag668-B5] Ashburner M , Ball CA, Blake JA et al Gene Ontology: tool for the unification of biology. The Gene Ontology Consortium. Nat Genet 2000;25:25–9. 10.1038/75556 PMC3037419 10802651

[btag668-B6] Audia JE , Campbell RM. Histone modifications and cancer. Cold Spring Harb Perspect Biol 2016; 8: a019521. 10.1101/cshperspect.a019521 PMC4817802 27037415

[btag668-B7] Babur Ö , Gönen M, Aksoy BA et al Systematic identification of cancer driving signaling pathways based on mutual exclusivity of genomic alterations. Genome Biol 2015;16:45. 10.1186/s13059-015-0612-6 PMC4381444 25887147

[btag668-B8] Bailey MH , Tokheim C, Porta-Pardo E et al Comprehensive characterization of cancer driver genes and mutations. Cell 2018;173:371–85.e18. 10.1016/j.cell.2018.02.060 PMC6029450 29625053

[btag668-B9] Balogh KN , Templeton DJ, Cross JV. Macrophage Migration Inhibitory Factor protects cancer cells from immunogenic cell death and impairs anti-tumor immune responses. PLoS One 2018;13:e0197702. 10.1371/journal.pone.0197702 PMC5986154 29864117

[btag668-B10] Boerner TJ , Deems S, Furlani TR et al ACCESS: advancing innovation: NSF’s advanced cyberinfrastructure coordination ecosystem: services & support. In: Practice and Experience in Advanced Research Computing. New York, NY: ACM, 2023.

[btag668-B11] Cancer Genome Atlas Research Network, Comprehensive molecular profiling of lung adenocarcinoma. Nature 2014;511:543–50. 10.1038/nature13385 PMC4231481 25079552

[btag668-B12] Chakravarty D , Gao J, Phillips S et al Oncokb: a precision oncology knowledge base. JCO Precision Oncology 2017;1–16. 10.1200/PO.17.00011 PMC5586540 28890946

[btag668-B13] Chuang H-Y , Lee E, Liu Y-T et al Network-based classification of breast cancer metastasis. Mol Syst Biol 2007;3:140. 10.1038/msb4100180 PMC2063581 17940530

[btag668-B14] Ciriello G , Cerami E, Aksoy BA et al Using MEMo to discover mutual exclusivity modules in cancer. Curr Protoc Bioinformatics 2013a;Chapter 8:8.17.1–12. 10.1002/0471250953.bi0817s41 PMC5563973 23504936

[btag668-B15] Ciriello G , Miller ML, Aksoy BA et al Emerging landscape of oncogenic signatures across human cancers. Nat Genet 2013b;45:1127–33. 10.1038/ng.2762 PMC4320046 24071851

[btag668-B16] Cooper WA , Lam D, O’toole SA et al Molecular biology of lung cancer. J Thorac Dis 2013;5:S479–90. 10.3978/j.issn.2072-1439.2013.08.03 PMC3804875 24163741

[btag668-B17] De Palma M , Hanahan D. Milestones in tumor vascularization and its therapeutic targeting. Nat Cancer 2024;5:827–43. 10.1038/s43018-024-00780-7 38918437

[btag668-B18] DepMap B. DepMap 24Q4 Public. 2024. 10.25452/FIGSHARE.PLUS.27993248.V1

[btag668-B19] Díaz-Gay M , Vangara R, Barnes M et al Assigning mutational signatures to individual samples and individual somatic mutations with SigProfilerAssignment. Bioinformatics 2023;39:btad756. 10.1093/bioinformatics/btad756 PMC10746860 38096571

[btag668-B20] Fearon ER , Vogelstein B. A genetic model for colorectal tumorigenesis. Cell 1990;61:759–67. 10.1016/0092-8674(90)90186-i 2188735

[btag668-B21] Friedman J , Hastie T, Tibshirani R. Regularization paths for generalized linear models via coordinate descent. J Stat Soft 2010;33:22. 10.18637/jss.v033.i01 PMC2929880 20808728

[btag668-B22] Garraway LA , Lander ES. Lessons from the cancer genome. Cell 2013;153:17–37. 10.1016/j.cell.2013.03.002 23540688

[btag668-B23] Gillette MA , Satpathy S, Cao S et al Proteogenomic characterization reveals therapeutic vulnerabilities in lung adenocarcinoma. Cell 2020;182:200–25.e35. 10.1016/j.cell.2020.06.013 PMC7373300 32649874

[btag668-B25] Hanahan D , Weinberg RA. Hallmarks of cancer: the next generation. Cell 2011;144:646–74. 10.1016/j.cell.2011.02.013 21376230

[btag668-B26] Hofree M , Shen JP, Carter H et al Network-based stratification of tumor mutations. Nat Methods 2013;10:1108–15. 10.1038/nmeth.2651 PMC3866081 24037242

[btag668-B27] Ideker T , Ozier O, Schwikowski B et al Discovering regulatory and signalling circuits in molecular interaction networks. Bioinformatics 2002;18 Suppl 1:S233–40. 10.1093/bioinformatics/18.suppl_1.s233 12169552

[btag668-B28] Jassal B , Matthews L, Viteri G et al The reactome pathway knowledgebase. Nucleic Acids Res 2020;48:D498–503. 10.1093/nar/gkz1031 PMC7145712 31691815

[btag668-B29] Kajiwara Y , Akram A, Katsel P et al FE65 binds Teashirt, inhibiting expression of the primate-specific caspase-4. PLoS One 2009;4:e5071. 10.1371/journal.pone.0005071 PMC2660419 19343227

[btag668-B30] Kim M , Park J, Bouhaddou M et al A protein interaction landscape of breast cancer. Science 2021;374:eabf3066. 10.1126/science.abf3066 PMC9040556 34591612

[btag668-B31] Kong J , Zhao X, Singhal A et al Prediction of immunotherapy response using mutations to cancer protein assemblies. Sci Adv 2024;10:eado9746. 10.1126/sciadv.ado9746 PMC11414719 39303028

[btag668-B32] Kundra R , Zhang H, Sheridan R et al OncoTree: a cancer classification system for precision oncology. JCO Clin Cancer Inform 2021;5:221–30. 10.1200/CCI.20.00108 PMC8240791 33625877

[btag668-B33] Lawrence MS , Stojanov P, Polak P et al Mutational heterogeneity in cancer and the search for new cancer-associated genes. Nature 2013;499:214–8. 10.1038/nature12213 PMC3919509 23770567

[btag668-B34] Leiserson MDM , Vandin F, Wu H-T et al Pan-cancer network analysis identifies combinations of rare somatic mutations across pathways and protein complexes. Nat Genet 2015;47:106–14. 10.1038/ng.3168 PMC4444046 25501392

[btag668-B35] Li Y , Porta-Pardo E, Tokheim C et al Pan-cancer proteogenomics connects oncogenic drivers to functional states. Cell 2023;186:3921–44.e25. 10.1016/j.cell.2023.07.014 37582357

[btag668-B36] Li Z , Yang Q, Xu L et al Adasemb: an adaptive knowledge-driven deep learning framework integrating cancer protein assemblies for predicting PI3Kα inhibitor response and resistance. Brief Bioinform 2025;26:bbaf510. 10.1093/bib/bbaf510 PMC12477610 41020523

[btag668-B37] Loshchilov I , Hutter F. SGDR: Stochastic gradient descent with warm restarts. *arXiv [csLG]* 2016. 10.48550/ARXIV.1608.03983, preprint: not peer reviewed.

[btag668-B38] Martincorena I , Raine KM, Gerstung M et al Universal patterns of selection in cancer and somatic tissues. Cell 2017;171:1029–41.e21. 10.1016/j.cell.2017.09.042 PMC5720395 29056346

[btag668-B39] Meyers RM , Bryan JG, McFarland JM et al Computational correction of copy number effect improves specificity of CRISPR-Cas9 essentiality screens in cancer cells. Nat Genet 2017;49:1779–84. 10.1038/ng.3984 PMC5709193 29083409

[btag668-B40] Mularoni L , Sabarinathan R, Deu-Pons J et al OncodriveFML: a general framework to identify coding and non-coding regions with cancer driver mutations. Genome Biol 2016;17:128. 10.1186/s13059-016-0994-0 PMC4910259 27311963

[btag668-B41] Otlu B , Alexandrov LB. Evaluating topography of mutational signatures with SigProfilerTopography. Genome Biol 2025;26:134. 10.1186/s13059-025-03612-8 PMC12093824 40394581

[btag668-B42] Otlu B , Díaz-Gay M, Vermes I et al Topography of mutational signatures in human cancer. Cell Rep 2023;42:112930. 10.1016/j.celrep.2023.112930 PMC10507738 37540596

[btag668-B43] Paczkowska M , Barenboim J, Sintupisut N et al Integrative pathway enrichment analysis of multivariate omics data. Nat Commun 2020;11:735. 10.1038/s41467-019-13983-9 PMC7002665 32024846

[btag668-B44] Park S , Silva E, Singhal A et al A deep learning model of tumor cell architecture elucidates response and resistance to CDK4/6 inhibitors. Nat Cancer 2024;5:996–1009. 10.1038/s43018-024-00740-1 PMC11286358 38443662

[btag668-B45] Pickup MW , Mouw JK, Weaver VM. The extracellular matrix modulates the hallmarks of cancer. EMBO Rep 2014;15:1243–53. 10.15252/embr.201439246 PMC4264927 25381661

[btag668-B46] Repana D , Nulsen J, Dressler L et al The Network of Cancer Genes (NCG): a comprehensive catalogue of known and candidate cancer genes from cancer sequencing screens. Genome Biol 2019;20:1. 10.1186/s13059-018-1612-0 PMC6317252 30606230

[btag668-B47] Reyna MA , Haan D, Paczkowska M et al Pathway and network analysis of more than 2500 whole cancer genomes. Nat Commun 2020;11:729. 10.1038/s41467-020-14367-0 PMC7002574 32024854

[btag668-B48] Reyna MA , Leiserson MDM, Raphael BJ. Hierarchical HotNet: identifying hierarchies of altered subnetworks. Bioinformatics 2018; 34:i972–980. 10.1093/bioinformatics/bty613 PMC6129270 30423088

[btag668-B49] Seal RL , Braschi B, Gray K et al Genenames.Org: the HGNC resources in 2023. Nucleic Acids Res 2023;51:D1003–9. 10.1093/nar/gkac888 PMC9825485 36243972

[btag668-B50] Sondka Z , Dhir NB, Carvalho-Silva D et al COSMIC: a curated database of somatic variants and clinical data for cancer. Nucleic Acids Res 2024;52:D1210–7. 10.1093/nar/gkad986 PMC10767972 38183204

[btag668-B51] Steele CD , Abbasi A, Islam SMA et al Signatures of copy number alterations in human cancer. Nature 2022;606:984–91. 10.1038/s41586-022-04738-6 PMC9242861 35705804

[btag668-B52] Strande S , Cai H, Tatineni M et al Expanse: computing without boundaries: architecture, deployment, and early operations experiences of a supercomputer designed for the rapid evolution in science and engineering. In: Practice and Experience in Advanced Research Computing. New York, NY: ACM, 2021.

[btag668-B53] Subramanian A , Tamayo P, Mootha VK et al Gene set enrichment analysis: a knowledge-based approach for interpreting genome-wide expression profiles. Proc Natl Acad Sci U S A 2005;102:15545–50. 10.1073/pnas.0506580102 PMC1239896 16199517

[btag668-B54] Swaney DL , Ramms DJ, Wang Z et al A protein network map of head and neck cancer reveals PIK3CA mutant drug sensitivity. Science 2021; 374:eabf2911. 10.1126/science.abf2911 PMC9005332 34591642

[btag668-B55] Tokheim C , Karchin R. CHASMplus reveals the scope of somatic missense mutations driving human cancers. Cell Syst 2019;9:9–23.e8. 10.1016/j.cels.2019.05.005 PMC6857794 31202631

[btag668-B56] Tsherniak A , Vazquez F, Montgomery PG et al Defining a cancer dependency map. Cell 2017;170:564–76.e16. 10.1016/j.cell.2017.06.010 PMC5667678 28753430

[btag668-B57] Tsitsiridis G , Steinkamp R, Giurgiu M et al CORUM: the comprehensive resource of mammalian protein complexes-2022. Nucleic Acids Res 2023;51:D539–45. 10.1093/nar/gkac1015 PMC9825459 36382402

[btag668-B24] van de Haar J , Canisius S, Yu MK et al Identifying epistasis in cancer genomes: a delicate affair. Cell 2019;177:1375–83. 10.1016/j.cell.2019.05.005 PMC6816465 31150618

[btag668-B58] Vandin F , Upfal E, Raphael BJ. Algorithms for detecting significantly mutated pathways in cancer. J Comput Biol 2011;18:507–22. 10.1089/cmb.2010.0265 21385051

[btag668-B59] Wendl MC , Wallis JW, Lin L et al PathScan: a tool for discerning mutational significance in groups of putative cancer genes. Bioinformatics 2011;27:1595–602. 10.1093/bioinformatics/btr193 PMC3106187 21498403

[btag668-B60] Yang L , Chen R, Goodison S et al An efficient and effective method to identify significantly perturbed subnetworks in cancer. Nat Comput Sci 2021;1:79–88. 10.1038/s43588-020-00009-4 PMC10284573 37346964

[btag668-B61] Zhao X , Singhal A, Park S et al Cancer mutations converge on a collection of protein assemblies to predict resistance to replication stress. Cancer Discov 2024;14:508–23. 10.1158/2159-8290.CD-23-0641 PMC10905674 38236062

[btag668-B62] Zheng F , Kelly MR, Ramms DJ et al Interpretation of cancer mutations using a multiscale map of protein systems. Science 2021;374:eabf3067. 10.1126/science.abf3067 PMC9126298 34591613

[btag668-B63] Zúñiga L , Cayo A, González W et al Potassium channels as a target for cancer therapy: current perspectives. Onco Targets Ther 2022;15:783–97. 10.2147/OTT.S326614 PMC9309325 35899081

