# Supplementary material for: Identifying multigenic modules under selection in the tumor genome
Source: Bioinformatics. 2026 Sep 9;42(9):btag668. doi: 10.1093/bioinformatics/btag668 (PMC13615650; doi:10.1093/bioinformatics/btag668)
Supplement: Supplementary file 1 [file btag668_supplementary_data.zip › Kelly_et_al_Supplemental_Material.docx]

# Supplementary Material

## Supplementary Methods

Output module maps were derived from the following sources. “Ciriello” : reference [(Ciriello *et al.* 2013a)](https://paperpile.com/c/eCw8k2/5PB4V) from the Supplementary Table [ovarian cancer, glioblastoma]; “Paczkowsca” : reference [(Paczkowska *et al.* 2020)](https://paperpile.com/c/eCw8k2/CUfgn), “PCAWG consensus” models from CDS [pan-cancer]; “Reyna” : reference [(Reyna *et al.* 2020)](https://paperpile.com/c/eCw8k2/fqtpI), Supplementary Table 24 [Pan-cancer]; “Leiserson” : reference [(Leiserson *et al.* 2015)](https://paperpile.com/c/eCw8k2/Ni4zP), Supplementary Table 19 [LUAD]; “Yang” : reference [(Yang *et al.* 2021)](https://paperpile.com/c/eCw8k2/tkitw) Supplementary Table 1 (breast cancer); “Li” : [(Li *et al.* 2023)](https://paperpile.com/c/eCw8k2/SIPDs) Supplementary Table S5E [pan-cancer].

# SUPPLEMENTARY FIGURES

**Supplementary Figure S1 CanSRMaPP model architecture.** **A** Schematic of the CanSRMaPP model. Rectangles indicate vectors, matrices, and tensors of indicated dimensions. Expon: exponential probability distribution function, wts.: weights, “mut”: point mutation (SNPs and Indels), “fus”: fusion, “gain”: copy gain, “loss”: copy loss. **B** Plate-notation diagram of the CanSRMaPP model. *Hyperparameters* α: selection:alteration distribution concentration, λ_wv_: rate constant for selection weight prior probability distribution, λ_z_: rate constant for background feature weight prior distribution; *indices* *g*: gene, *m*: module, *b*: background feature, τ: alteration type (as in A); *parameters* w_g_: single-gene selection weight, v_m_: module selection weight; z_b_: genomic background weight, e_τ,g_: partition of selection pressure on gene *g* into alteration type *τ*, *p*_τ,g_: inferred probability of genetic alteration; *predefined values* *h_g,m_*: gene-module membership, *j_τ,g,b_*: background feature relationship to gene alteration, *r_τ,g,g_*: synteny relationships, *y_τ,g_*: observed alteration counts in cohort. Symbols are described with greater detail in Methods. **C** Optimization of hyperparameters λ_𝐰_ and $\alpha$. A set of Pareto-optimal points were identified by minimizing the averaged posterior probability of 5 randomized maps (*x* axis) and maximizing the posterior probability using the NeSTv0 map (*y* axis), both relative to posterior probability under a null map. The point corresponding to chosen hyperparameters is circled in red. **D** Precision and recall of curated driver alterations at different hyperparameter combinations tested.

**Supplementary Figure S2 Assessing CanSRMaPP performance.** **A** Scatterplot of observed occurrences of gene-level alterations observed in the TCGA-LUAD cohort and predicted by CanSRMaPP (total 498 tumors). **B** Occurrences of gene-level alterations in the CPTAC-LUAD cohort, plotted as in A. **C** Heatmap comparing genes identified in each map or list. Black cells indicate that a gene was incorporated in the lists; only genes identified by at least two lists are plotted. Top: total appearances of each gene; right : total number of genes associated with each map or list. TCGA LUAD : ref. [(Cancer Genome Atlas Research Network 2014)(The Cancer Genome Atlas Research Network 2014)](https://paperpile.com/c/eCw8k2/rnbEw), Supplementary Tables 4,6, and 10. COSMIC v100 NSCLC: [COSMIC v100](https://cancer.sanger.ac.uk/cosmic/download/cosmic/v100/cancergenecensus) release; all somatic mutations annotated with NSCLC, “lung” or “luad”, plus *CDKN2A* and *MYC*. Leiserson15 LUAD: Leiserson et al, 2015[(Leiserson *et al.* 2015)](https://paperpile.com/c/eCw8k2/Ni4zP), Supplementary Table 19. Bailey18lung|pancan: Bailey et al, 2018[(Bailey *et al.* 2018)](https://paperpile.com/c/eCw8k2/IaMtb); Supplementary table S1 annotated to “LUAD” or “PANCAN” ; modules from Supplementary Table S2. NeSTv1 LUAD: under selection in LUAD from ([ccmi.org/nest](http://ccmi.org/nest)), all genes appearing in Supplementary Table S8[(Zheng *et al.* 2021)](https://paperpile.com/c/eCw8k2/RQ5P1). **D** log_2_-fold error of the CanSRMaPP model for events in at least 3 gene lists but not under selection, plotted against the alteration rate of that event in the TCGA LUAD cohort (498 tumors). The dark gray patch contains alterations predicted within 2-fold error despite lack of selection effects (gray wedge in 2H) ; the light gray patch contains other alterations occurring in <4% of tumors (light wedge in 2H).

**Supplementary Figure S3 Applying CanSRMaPP with different module maps. A** Pearson correlation of predicted vs. observed genetic alterations in the TCGA-LUAD (green) and CPTAC-LUAD (gold) cohorts. **B** Comparison of posterior probabilities for models using the indicated module maps and randomized versions of those maps (**Methods**). **C** Number of features of each type included in each model (i.e. features with weights > 0). **D** Number of events under selection in each model. **E** Similarity of modules included in models using the indicated map. **F** Jaccard similarity of events under selection according to the models using the indicated map.

**Supplementary Figure S4 Applying CanSRMaPP to different tumor cohorts. A** Heatmap of features included of CanSRMaPP models of each tumor. Marginal totals (of 20 cohorts) are plotted at the top. **B** Total number of features included in each model. **C** Total number of tumors in each cohort. **D** Scatter plot of the mean events per tumor in each cohort vs. the number of features included in the model of that cohort.

**Supplementary Figure S5 CanSRMaPP-powered models of molecular phenotypes.** Distribution of Spearman correlations (ρ) of models of RPPA readouts using CanSRMaPP features or using genes from the indicated sources. Only the 291 readouts with at least one successful model are shown.

# DESCRIPTION OF SUPPLEMENTARY TABLES

**Supplementary Table 1 (**Supplementary_Table_1.xlsx) Description of mutational signatures used as genomic background features, and table of which cancer types incorporated those signatures as candidate features for model inclusion.

**Supplementary Table 2** (Supplementary_Table_2.xlsx) Results of hyperparameter optimization. Related to **Supplementary Figure S1C,D, Methods**.

**Supplementary Table 3** (Supplementary_Table_3.xlsx) maximum *a* *posteriori* estimates for all features in all non-control models. Related to **Figures 2-4, Supplementary Figures S2-4, Methods.**

**Supplementary Table 4** (Supplementary_Table_5.xlsx) FDRs for selection on genetic alterations for all non-control models. Related to **Figures 2-4, Supplementary Figures S2-4, Methods.**

**Supplementary Table 5** (Supplementary_Table_5.xlsx) Results of all RPPA modeling attempts. Related to **Figure 5, Supplementary Figure S5**.
